# Deep Inguinal Lymph Node Metastases Can Predict Pelvic Lymph Node Metastases and Prognosis in Penile Squamous Cell Carcinoma

**DOI:** 10.3389/fonc.2021.715799

**Published:** 2021-09-15

**Authors:** Zhenyu Yang, Xingliang Tan, Yanjun Wang, Yuantao Zou, Dong Chen, Zhiming Wu, Zhuowei Liu, Yonghong Li, Zike Qin, Hui Han, Fangjian Zhou, Kai Yao

**Affiliations:** ^1^Department of Urology, Sun Yat-sen University Cancer Center, Guangzhou, China; ^2^State Key Laboratory of Oncology in South China, Sun Yat-sen University Cancer Center, Guangzhou, China; ^3^Collaborative Innovation Center for Cancer Medicine, Sun Yat-sen University Cancer Center, Guangzhou, China

**Keywords:** lymph node dissection, neoplasm metastasis, penile neoplasms, prognosis, staging

## Abstract

**Objectives:**

To evaluate the relationship between deep inguinal lymph node metastasis (ILNM) and pelvic lymph node metastasis (PLNM) and explore the prognostic value of deep ILNM in penile squamous cell carcinoma (PSCC).

**Materials and Methods:**

The records of 189 patients with ILNM treated for PSCC were analysed retrospectively. Logistic regression models were used to test for predictors of PLNM. Cox regression was performed in univariable and multivariable analyses of cancer-specific survival (CSS). CSS was compared using Kaplan-Meier analyses and log rank tests.

**Results:**

PLNM were observed in 53 cases (28.0%). According to logistic regression models, only deep ILNM (OR 9.72, p<0.001) and number (≥3) of metastatic inguinal lymph nodes (ILNs) (OR 2.36, p=0.03) were independent predictors of PLNM. The incidences of PLNM were 18% and 19% with negative deep ILNM and extranodal extension (ENE); and 76% and 42% with positive deep ILNM and ENE, respectively. The accuracy of deep ILNM, ENE, bilateral involvement and number (≥3) of ILNMs for predicting PLNM were 81.0%, 65.6%, 63.5% and 67.2%, respectively. The CSS was significantly different in patients with positive and negative deep ILNM (median 1.7 years *vs* not reached, p<0.01). Patients who presented with deep ILNM had worse CSS (median 3.8 years *vs* not reached, p<0.01) in those with negative PLNs.

**Conclusions:**

Deep ILNM is the most accurate factor for predicting PLNM in PSCC according to our data. We recommend that patients with deep ILNM should be referred for pelvic lymph node dissection. Involvement of deep ILNs indicates poor prognosis. We propose that patients with metastases of deep ILNs may be staged as pN3.

## Introduction

Lymph node metastasis (LNM) is the major prognostic factor for survival of penile squamous cell carcinoma (PSCC) (1). Regional lymph nodes (LNs) of the penis include inguinal and pelvic nodes. Therapeutic radical inguinal lymph node dissection (ILND) and pelvic lymph node dissection (PLND) are important treatments for PSCC (2). Inguinal lymph nodes (ILNs) consist of superficial and deep nodes, and both superficial and deep ILNs should be removed in complete ILND (3). Lymphatic drainage of the penis is to the superficial and deep ILNs and to the pelvic lymph nodes (PLNs) (4). Thus, PSCC metastasizes in a stepwise fashion from the primary tumor to the ILNs and PLNs (4, 5).

PLND is not recommended when metastasis of deep ILNM is observed according to latest guidelines on penile cancer, although it was recommended before 2014 (6, 7). Additionally, superficial and deep ILNs were not distinguished according to guidelines. The recommendation is mainly based on a study by Leijte et al. (8). However, the relationship between deep inguinal LNM (ILNM) and pelvic LNM (PLNM) and the prognosis of deep ILNM were not evaluated in that study. Interestingly, ILND is routinely performed in patients with groin LNM from melanoma. PLND should be performed if deep ILNs are positive according to NCCN guidelines for cutaneous melanoma (9). The tumor status of deep ILNs is associated with PLNM and survival in melanoma and vulvar cancer (10, 11).

Few studies with small series of cases have evaluated the relationship between the tumor status of deep ILNs and PLNs in penile cancer (12, 13), which showed that deep ILNM may be associated with PLNM. Unfortunately, data on the clinical characteristics of deep ILNM in penile cancer are still scarce. Thus, the tumor status of deep ILNs is ignored by the latest guidelines on penile cancer.

In this retrospective study, we aimed to assess whether deep ILNM is associated with PLNM and explore the prognostic value of deep ILNM in PSCC.

## Materials and Methods

### Study Population

After institutional review board and ethics committee approval was obtained, data were collected on patients in our institution with PSCC treated between January 2000 and June 2020. The informed consent was waived since the retrospective nature of this study. Patients were screened according to following inclusion criterions: 1) Pathologically confirmed PSCC; 2) Bilateral ILND were performed and pathologically confirmed with nodes metastases; 3) Deep inguinal lymphatic tissues were dissected separately; 4) Bilateral PLND were performed, or not performed but followed up with more than two years without evidence of PLNM. Patients who did not receive bilateral PLND were grouped with those without PLNM at histopathological evaluation (8, 14, 15). Patients who received neoadjuvant therapy or had less than 10 total ILNs removed without a fixed nodal mass were excluded.

### Indications and Surgical Technique

**Figure 1:**
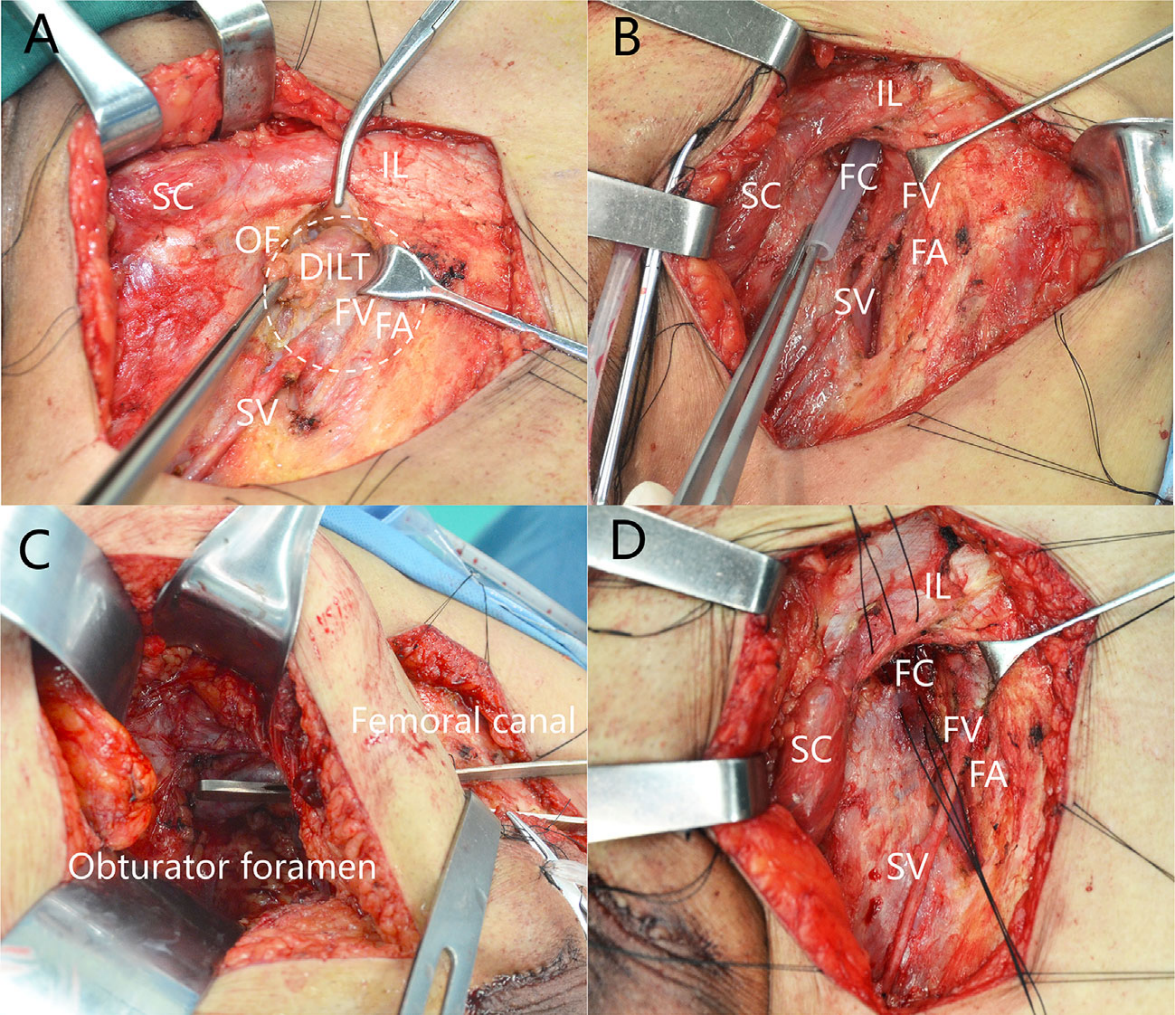
Deep inguinal lymph nodes dissection. **(A)** Position of deep inguinal lymph nodes. **(B)** The femoral canal is empty after removal of DILT. **(C)** Femoral canal communicates with obturator after removal of DILT and pelvic LNs. **(D)** Closing the femoral canal. DILT, deep inguinal lymphatic tissue; FA, femoral artery; FC, femoral canal; FV, femoral vein; IL, inguinal ligament; OF, oval fossa; SC, spermatic cord; SV, saphenous vein.

All the surgeries were performed by experienced surgeons. ILND was indicated according to established guidelines, which have been discussed previously (16, 17). After superficial nodes were removed, the cribriform fascia near the femoral canal was divided. Deep ILNs lying in the femoral canal medial to the femoral vein were dissected (**Figure 1A**). The femoral canal and obturator foramen can be communicated after removal of deep inguinal lymphatic tissue and PLNs (**Figures 1B, C**). The femoral canal should be closed after dissection of deep ILNs (**Figure 1D**) in cases of hernia.

It was controversial to perform PLND in penile cancer before 2009. The decision to perform PLND varied over time and at each institution (14, 18, 19). Thus, only some patients at our institution received PLND before 2009. After that time, synchronous or secondary PLND was indicated for patients if two or more positive ILNs, ENE, or suspicious pelvic imaging were found following radical ILND. PLND consisted of the removal of common iliac, external iliac, internal iliac, obturator and presacral LNs, which was described previously (15, 20).

### Staging, Node Count and Follow-Up

All LN specimens were reviewed by two dedicated uropathologists at our institution. After pathological review, clinical and pathological nodal categories were determined according to the 8th edition AJCC staging system for penile cancer. A fixed or gross nodal mass was counted as one LN regardless of size and ENE (14, 21). Deep inguinal LNs were counted as zero and categorized as negative if no LNs were identified in deep inguinal lymphatic tissue. Previous studies revealed that having more than 2 positive ILNs was an independent predictive factor for PLNM (14, 19). Thus, a positive ILNs cutoff of 3 or greater was set in this study for the logistic regression analyses. Follow-up, including physical examination, ultrasound, CT scan or MRI, was performed every 3 months for the first 2 years, every 6 months for the next 3 years, and annually thereafter, for all patients enrolled.

### Statistical Analysis

Mann-Whitney *U* and chi-square tests were used to compare continuous and categorical variables, respectively. Univariate and multivariable logistic regression models were used to determine independent predictors for PLNM. Univariable and multivariable Cox regression models were used to test factors of cancer-specific survival (CSS). The Kaplan-Meier method was used to explore CSS rates, and differences were assessed using the log rank test. All reported *p* values are two-sided, with statistical significance considered at *p*<0.05. All statistical analyses were performed with SPSS version 22 (SPSS Inc., Chicago, IL, USA) and R statistical package version 3.6.3 (R Project for Statistical Computing, www.r-project.org).

## Results

### Patient Characteristics

A total of 632 patients received penectomies during the period we analysed, and 189 of them were eligible and included in this study. PLNM was confirmed histopathologically based on PLND in 53 (28.0%) patients. A total of 128 (67.7%) patients received bilateral PLND. 61 patients (32.3%) who did not receive bilateral PLND with negative follow-up were grouped with negative PLNM. Deep ILNs were not identified in 95 (25.1%) groins. Clinical and pathological characteristics are summarized in **Table 1**. Mean follow-up was 4.1 (IQR 1.5-5.9) years.

**Table 1:** Clinical and pathological characteristics of the 189 patients with penile SCC.

Characteristics	Overall	ILNM only	ILNM and PLNM	*p* value
Number of patients	189	136	53	–
Age, Median (IQR)	52 (44–62)	51 (43-59)	55 (47-67)	0.423^
Treatment of primary tumor, n (%)				0.714*
Circumcision	16 (8.5%)	10 (7.4%)	6 (11.3%)	
Partial penectomy	137 (72.5%)	100 (73.5%)	37 (69.8%)	
Total penectomy	25 (13.2%)	19 (14.0%)	6 (11.3%)	
Unknow	11 (5.8%)	7 (5.1%)	4 (7.5%)	
pT stage, n (%)				0.428*
≤pT1	79 (41.8%)	62 (45.6%)	17 (32.1%)	
pT2	56 (29.6%)	37 (27.2%)	19 (35.8%)	
pT3	39 (20.6%)	26 (19.1%)	13 (24.5%)	
pT4	5 (2.6%)	3 (2.2%)	2 (3.8%)	
pTx	10 (5.3%)	8 (5.9%)	2 (3.8%)	
Tumor grade, n (%)				0.229*
G1	73 (38.6%)	58 (42.6%)	15 (28.3%)	
G2	88 (46.6%)	61 (44.9%)	27 (50.9%)	
≥G3	19 (10.1%)	11 (8.1%)	8 (15.1%)	
Gx	9 (4.8%)	6 (4.4%)	3 (5.7%)	
No. of ILNs removed, Median (IQR)	24 (17-29)	24 (18-30)	20 (16-27)	0.058^
No. of deep ILNs removed, Median (IQR)	3 (2-4)	3 (2-4)	3 (2-4)	0.641^
No. of PLNs removed, Median (IQR)	20 (14-28)	20 (14-30)	21 (14-27)	0.827^
No. of positive ILNs, Median (IQR)	2 (1-4)	2 (1-3)	4 (2-6)	<0.001^
Deep ILNs, n (%)				<0.001*
Positive	33 (17.5%)	8 (5.9%)	25 (47.2%)	
Negative	156 (82.5%)	128 (94.1%)	28 (52.8%)	
Extranodal extension of ILNs, n (%)				0.001*
Present	74 (39.2%)	43 (31.6%)	31 (58.5%)	
Absent	115 (60.8%)	93 (68.4%)	22 (41.5%)	
Diameter of ILN, n (%)				0.101*
<30 mm	88 (46.6%)	68 (50.0%)	20 (37.7%)	
≥30 mm	92 (48.7%)	60 (44.1%)	32 (60.4%)	
Unknow	9 (4.8%)	8 (5.9%)	1 (1.9%)	
Side involvement of ILNs, n (%)				0.002*
Bilateral	80 (42.3%)	48 (35.3%)	32 (60.4%)	
Unilateral	109 (57.7%)	88 (64.7%)	21 (40.6%)	
Lymphovascular invasion, n (%)				0.087*
Present	66 (34.9%)	41 (30.1%)	25 (47.2%)	
Absent	118 (62.4%)	91 (66.9%)	27 (50.9%)	
Unknow	5 (2.6%)	4 (2.9%)	1 (1.9%)	
Adjuvant therapy, n (%)				0.01*
Positive	98 (51.9%)	62 (45.6%)	32 (61.4%)	
Negative	75 (39.7%)	66 (48.5%)	13 (24.5%)	
Unknow	16 (8.5%)	8 (5.9%)	8 (15.1%)	

SCC, squamous cell carcinoma; IQR, interquartile range; ILN, inguinal lymph nodes; ILNM, inguinal lymph node metastases; PLN, pelvic lymph nodes; PLNM, pelvic lymph node metastases.

*Chi-square test; ^Mann-Whitney’s test.

### Predicting PLNM by Pathological Characteristics of ILNs

Patients who presented with PLNM had a significantly higher incidence of deep ILNM (47.2% *vs* 5.9%, *p*<0.001), ENE (58.5% *vs* 31.6%, *p*=0.001), bilateral involvement of ILNs (60.4% *vs* 35.3%, *p*=0.002), and a greater number of positive ILNs (median 4 *vs* 2, *p*<0.001) than those with negative PLNs (**Table 1**). On univariable logistic regression analyses, deep ILNM (OR 14.29, *p*<0.001), ENE (OR 3.05, *p*=0.001), 3 or more positive ILNs (OR 4.52, *p*<0.001) and bilateral involvement (OR 2.79, *p*=0.002) were significant predictors of PLNM (**Table 2**). Only 2 factors (deep ILNM and 3 or more positive ILNs) emerged as independent predictors of PLNM in the multivariable logistic regression models (**Table 2**). When patients were classified based on the number of positive ILNs, the incidence of PLNM increased in parallel with the number of positive ILNs for patients with positive and negative ENE (**Supplementary Table 1**). This was also observed in patients with bilateral and unilateral involvement. However, the incidence of PLNM was relatively lower in patients with negative deep ILNs than in those with positive deep ILNs. PLNM incidences were consistently high in patients with positive deep ILNs (**Supplementary Table 1**).

**Table 2:** Univariable and multivariable logistic regression analysis predicting PLNM by inguinal lymph node characteristics.

Predictors	Univariable		Multivariable	
	OR (95% CI)	*p* value	OR (95% CI)	*p* value
+ Deep ILN (no *vs* yes)	14.29 (5.84-34.96)	<0.001	9.72 (3.77-25.08)	<0.001
+ ENE (no *vs* yes)	3.05 (1.58-5.87)	0.001	–	–
>2 Positive ILNs (no *vs* yes)	4.52 (2.28-8.98)	<0.001	2.36 (1.09-5.13)	0.03
Bilateral involvement (no *vs* yes)	2.79 (1.45-5.37)	0.002	–	–
>30mm diameter of metastatic (no *vs* yes)	1.81 (0.94-3.50)	0.076	–	–

PLNM, pelvic lymph node metastasis; ILN, inguinal lymph node; ENE, extranodal extension.

**Figure 2:**
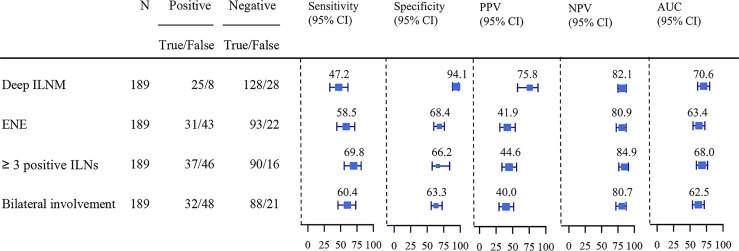
Sensitivity, specificity, PPV, NPV and AUC of ILNs characteristics predicting PLNM. PLNM, pelvic lymph node metastasis; ILNM, inguinal lymph node metastasis; ENE, extranodal extension; AUC, Area under the curve; PPV, positive predictive value; NPV, negative predictive value.

The predictive values of ILNs characteristics for predicting PLNM are shown in **Figure 2**. The specificity (94.1%) and positive predictive value (PPV) (75.8%) of deep ILNM were higher than those of any other predictor, although the sensitivity (47.2%) was relatively low. The negative predictive value (NPV) was comparable for all predictors. ENE, ≥3 positive ILNs and bilateral involvement had similar predictive values. The accuracy (81.0% *vs* 65.6% *vs* 57.2% *vs* 63.5%) (true positive and true negative) and the area under the curve (AUC) (0.71) of deep ILNM were better than those of any other factors.

### Survival Analysis

**Figure 3:**
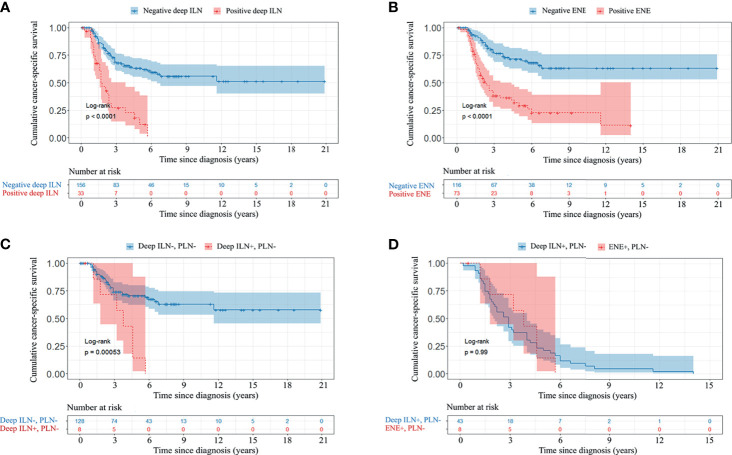
Kaplan-Meier CSS curve of patients with different ILN and PLN characteristics. **(A)**, deep ILN. **(B)**, ENE. **(C)**, deep ILNM without PLNM. **(D)**, deep ILNM and ENE without PLNM. CSS, cancer-specific survival; PLN, pelvic lymph node; ILN, inguinal lymph node; ENE, extranodal extension.

The median CSS was 2.9 (IQR 1.5-5.9) years. Univariable Cox regression analyses showed that deep ILNM, ENE, bilateral involvement, 3 or more positive inguinal LNs and diameter of metastatic ILNs were significant prognostic factors of CSS (**Table 3**). In multivariable Cox regression analyses, deep ILNM (HR 2.07, *p* =0.007), ENE (HR 2.72, *p <*0.001) and bilateral involvement (HR 2.37, *p <*0.001) remained independent prognostic factors for CSS (**Table 3**). The CSS was significantly different in patients with positive and negative deep ILNM (median 1.7 years *vs* not reached) and in those with positive and negative ENE (median 2.3 years *vs* not reached) (**Figures 3A, B**). Patients who presented with deep ILNM still had worse CSS (median 3.8 years *vs* not reached) in those with negative PLNs. (**Figure 3C**). CSS was similar between those with deep ILNM and ENE in patients with PLNM (median 1.6 *vs* 1.6 years) (**Supplementary Figure 1**). However, considering patients with negative PLNs, there was still no significant difference in CSS between patients with deep ILNM and ENE (median 3.8 *vs* 2.9 years) (**Figure 3D**).

**Table 3:** Univariable and multivariable Cox regression analyses of variables on CSS.

Prognostic variables	Univariable		Multivariable	
	HR (95% CI)	*P* value	HR (95% CI)	*P* value
+ Deep ILN (no *vs* yes)	4.08 (2.51-6.64)	<0.001	2.07 (1.22-3.50)	0.007
+ ENE (no *vs* yes)	3.76 (2.38-5.99)	<0.001	2.72 (1.66-4.45)	<0.001
>2 Positive ILNs (no *vs* yes)	3.11 (1.98-4.89)	<0.001	2.37 (1.49-3.78)	<0.001
Bilateral involvement (no *vs* yes)	3.38 (2.14-5.34)	<0.001	–	–
>30mm diameter of metastatic (no *vs* yes)	1.63 (1.04-2.57)	0.035	–	–

CSS, cancer-specific survival; ILNM, inguinal lymph node; ENE, extranodal extension.

## Discussion

PLNM is a major prognostic factor in PSCC patients, which results in a 5-year survival of 12%~33% (14, 19, 22, 23). Approximately one-third of patients with ILNM from PSCC have PLNM (14). Thus, it is important to identify patients with PLNM early. Conventional images, including CT and MRI, are limited in identifying patients with PLNM and result in low sensitivity. Thus, assessment of the pathological characteristics of ILNs is indicated for PLND (6). In this retrospective study, we analyzed whether metastases of deep ILNs can indicate involvement of PLNs.

Previous studies have shown that the histopathological characteristics of ILNs, including the number of positive nodes, tumor grade of the involved nodes, lymph node density, diameter, and ENE, are predictive factors associated with PLNM (14, 18, 19). However, these studies ignored the tumor status of deep ILNs. Although lymphatic drainage of the penis occurs to the superficial and deep ILNs and to the PLNs sequentially; PSCC metastasizes along a similar stepwise pathway (4, 5). Thus, it is important to evaluate the status of deep ILNs in PSCC. We usually dissected superficial and deep ILNs separately in our center in the last 20 years, and we observed that metastases of PLNs usually occurred when deep ILNs were involved. Therefore, the significance of deep ILNM needs to be evaluated due to the metastatic pathway of PSCC.

Leijte et al. (8) previously proposed that deep ILNM should be removed from pN3 cases and the distinction between superficial and deep ILNs should be eliminated, because they found that superficial and deep ILNs cannot be easily distinguished (8). These recommendations were later adopted by the AJCC staging system and guidelines on penile cancer. However, the relationship between deep ILNM and PLNM, and the prognostic value of deep ILNM, were not analysed in that study. Furthermore, superficial and deep ILNs can be distinguished during surgery according to our experience, as their anatomical positions are totally different (**Figure 1A**) (3). The indications for PLND in the latest guidelines on penile cancer were mainly based on a study by Lughezzani et al. (14). However, the relationship between metastases of deep ILNs and metastases of PLNs was not analyzed in that study.

Although a few studies have evaluated the relationship between the tumor status of deep ILNs and PLNs, the number of cases in these studies is very small (24 and 30 patients with positive inguinal nodes, respectively) (12, 13). Our study shows that deep ILNM emerged as an independent predictor of PLNM. The specificity, PPV, accuracy and AUC of deep ILNM were higher than those of any other factors evaluated in this study. PLNM presented in 75% of patients with deep ILNM, which only occurred in 46%, 42%, and 40% of patients with ≥3 positive inguinal LNs, ENE, and bilateral involvement, respectively. Moreover, PLNM incidence was high in all patients with deep ILNM, regardless of the number of positive ILNs in our study, even in those with only 2 positive ILNs (75%). This may endorse the recommendation that patients with deep ILNM should receive PLND. Deep ILNM was not observed in patients with 1 positive inguinal LN. This may indicate that deep ILNs are not sentinel nodes. The sensitivity (47.2%) of deep ILNM for predicting PLNM is relatively low. This result suggests that negative deep ILNs cannot rule out PLNM. One possible explanation is that occult metastases of deep ILNs were not identified histopathologically; and another is that there may be other direct pathways of lymphatic drainage from superficial ILNs to PLNs, bypassing deep ILNs.

The prognostic value of deep ILNs in penile cancer was not evaluated previously. Notably, when all patients were considered, patients with positive deep ILNM had a significantly worse prognosis than those with negative ILNM in our study. This was also observed in patients with negative PLNM. Furthermore, the prognosis was similar between patients with positive deep ILNM and ENE in negative PLN patients. All these results indicate that deep ILNM is a prognostic factor associated with poor survival, which is similar to inguinal ENE (24). ENE is categorized as pN3 according to AJCC staging system, thus, metastases of deep ILNs may be categorized as pN3 according to our data. However, this proposal is not validated yet, and more data are required to verify it.

The retrospective nature and long interval of our series represents a potential limitation. However, All the surgeries were performed by experienced surgeons. Additionally, to the best of our knowledge, this study has the largest sample size evaluating the relationship between the tumor status of ILNs and PLNs. Therefore, large sample size, standardized and similar treatment strategy minimized the shortcomings of retrospective design. Penile cancer is rare, so long duration is required to collect enough cases. Previous studies focus on penile cancer even across longer period of time (14, 19). Also, prospective studies evaluating relationship of inguinal and pelvic metastases are unlikely, due to the low incidence of penile cancer.

There were 95 groins (25.1%) with no LNs identified in deep inguinal lymphatic tissue, which could be considered a limitation of our study. However, the average number of deep inguinal LNs dissected was 1.4 per groin, which is consistent with previous studies (11, 25). Inguinal regions of 19 male cadavers were dissected by de Carvalho et al. (25). Deep ILNs were not encountered in all cases, even though all cadavers were dissected carefully. Absence of deep inguinal LNs was also observed in a study by Zhu et al. (13). Several factors contribute to the lack of confirmation of deep ILNs. First, deep ILNs may be absent in some groins. In addition, deep ILNs may be missed during histopathological analyses due to their small size and number. Patients with deep ILNs not identified were categorized as negative in our study. This may lower the sensitivity of deep ILNs predicting PLNM. However, this does not change our opinion that patients with deep ILNM should receive PLND.

The inclusion of patients (32.3%, 61) who did not receive PLND may also be considered a limitation. However, these patients were followed up more than two years, and no pelvic metastases occurred. Virtually all metastases manifest within this period (8, 14, 15). The follow-up of some patients with negative PLNs was relatively short. However, all patients received standardized surgery. The recurrence rate was relatively low in our institution, which was reported previously (16, 17). Furthermore, bilateral, rather than unilateral, PLND was performed in our study, although the guidelines recommend that bilateral PLND is not necessary for all patients. Zargar-Shoshtari et al. (26) found that metastases can spread from ILNs on one side to the PLNs on the other side. This was also found in our previous study (20). As such, metastatic PLNs can be removed more completely by bilateral PLND.

There are other limitations to our data and findings. Patients who received neoadjuvant chemotherapy were excluded from our study. Though this avoided the impact of a therapy response from neoadjuvant chemotherapy, bias may have occurred as some advanced patients were not included. However, approximately 16% of patients can achieve a pathological complete response with neoadjuvant chemotherapy (27). It is difficult to distinguish patients with pathological complete response and absent pelvic metastases. Thus, patients who received neoadjuvant chemotherapy had to be excluded. Additionally, a bulky nodal mass was counted as one lymph node in our study. The true number of involved LNs is unknown in such cases. The number of positive ILNs remained statistically significant in the multivariable analysis, even though a bulky nodal mass was counted as one.

In conclusion, metastases of deep ILNs is the most accurate nodal feature predicting PLNM in PSCC according to our data. We recommend that patients with deep ILNM should receive PLND according to our findings, regardless of the number of positive ILNs and other histopathological characteristics of ILNs. Additionally, metastases of deep ILNs affect prognosis. We propose that patients with involvement of deep ILNs may be staged as pN3, but this proposal need to be verified in the future.

## Data Availability Statement

The raw data supporting the conclusions of this article will be made available by the authors, without undue reservation.

## Ethics Statement

The studies involving human participants were reviewed and approved by Institutional review board and ethical committee of Sun Yat-sen University Cancer Center. Written informed consent for participation was not required for this study in accordance with the national legislation and the institutional requirements.

## Author Contributions

ZY, HH, FZ, and KY designed this study. ZY drafted the manuscript. ZY, XT, YW, and YZ collected the data. ZY, DC, ZW, ZL YL, and ZQ analysed and interpreted the data, prepared the figures and tables for the manuscript. HH, FZ, and KY surprised this study. All authors contributed to the article and approved the submitted version.

## Funding

This study was supported by the Fundamental Research Funds for the Central Universities (No.19ykpy178), Natural Science Foundation of Guangdong Province (No. 2019A1515010197) and Sun Yat-sen University Cancer Center Medical scientist training program (No.14zxqk08).

## Conflict of Interest

The authors declare that the research was conducted in the absence of any commercial or financial relationships that could be construed as a potential conflict of interest.

## Publisher’s Note

All claims expressed in this article are solely those of the authors and do not necessarily represent those of their affiliated organizations, or those of the publisher, the editors and the reviewers. Any product that may be evaluated in this article, or claim that may be made by its manufacturer, is not guaranteed or endorsed by the publisher.

## Supplementary Material

Supplementary Table 1 Pelvic lymph node metastasis based on inguinal lymph node characteristics. ILN, inguinal lymph node; ENE, extranodal extension; PLNM, pelvic lymph node metastasis.

Supplementary Figure 1 Kaplan-Meier CSS curve of patients with deep ILNM, ENE and PLNM. CSS, cancer-specific survival; PLN, pelvic lymph node; ILN, inguinal lymph node; ENE, extranodal extension.

## Abbreviations

AUC, area under the curve; CSS, cancer-specific survival; ENE, extranodal extension; ILND, inguinal lymph node dissection; ILNM, inguinal lymph node metastasis; ILN, Inguinal lymph node; LNM, lymph node metastasis; LN, lymph node; NPV, negative predictive value; PLND, pelvic lymph node dissection; PLNM, pelvic lymph node metastasis; PLN, pelvic lymph node; PPV, positive predictive value; PSCC, penile squamous cell carcinoma.

## References

[1] Ficarra V Akduman B Bouchot O Palou J Tobias-Machado M. Prognostic Factors in Penile Cancer. Urology (2010) 76(2 Suppl 1):S66–73. doi: 10.1016/j.urology.2010.04.008 20691887

[2] Protzel C Alcaraz A Horenblas S Pizzocaro G Zlotta A Hakenberg OW. Lymphadenectomy in the Surgical Management of Penile Cancer. Eur Urol (2009) 55(5):1075–88. doi: 10.1016/j.eururo.2009.02.021 19264390

[3] Daseler EH Anson BJ Reimann AF. Radical Excision of the Inguinal and Iliac Lymph Glands; a Study Based Upon 450 Anatomical Dissections and Upon Supportive Clinical Observations. Surg Gynecol Obstet (1948) 87(6):679–94. 18120502

[4] Cabanas RM. An Approach for the Treatment of Penile Carcinoma. Cancer (1977) 39(2):456–66. doi: 10.1002/1097-0142(197702)39:2<456::aid-cncr2820390214>3.0.co;2-i 837331

[5] Leijte JA Valdes Olmos RA Nieweg OE Horenblas S. Anatomical Mapping of Lymphatic Drainage in Penile Carcinoma With SPECT-CT: Implications for the Extent of Inguinal Lymph Node Dissection. Eur Urol (2008) 54(4):885–90. doi: 10.1016/j.eururo.2008.04.094 18502024

[6] Hakenberg OW Minhas ES Necchi A Protzel C Watkin N Compérat E. EAU Guidelines on Penile Cancer 2020. European Association of Urology Guidelines 2020 Edition. Presented at the EAU Annual Congress Amsterdam 2020. Arnhem, The Netherlands: European Association of Urology Guidelines Office (2020).

[7] National Comprehensive Cancer Network. Penile Cancer (Version 1, 2021). (2021). Available at: https://www.nccn.org/professionals/physician_gls/pdf/cutaneous_melanoma.pdf. 10.6004/jnccn.2021.0028 PMC8375577 34214969

[8] Leijte JA Gallee M Antonini N Horenblas S. Evaluation of Current TNM Classification of Penile Carcinoma. J Urol (2008) 180(3):933–8. doi: 10.1016/j.juro.2008.05.011 18635216

[9] National Comprehensive Cancer Network. Cutaneous Melanoma (Version 3, 2020). (2020). Available at: https://www.nccn.org/professionals/physician_gls/pdf/cutaneous_melanoma.pdf.

[10] Essner R Scheri R Kavanagh M Torisu-Itakura H Wanek LA Morton DL. Surgical Management of the Groin Lymph Nodes in Melanoma in the Era of Sentinel Lymph Node Dissection. Arch Surg (2006) 141(9):877–82. doi: 10.1001/archsurg.141.9.877 16983031

[11] Tu H Sun P Gu HF Zhang XK Huang H Wan T. Clinical Significance and Prognostic Value of Femoral Lymph Node Metastasis in FIGO Stage III Vulvar Carcinoma. Eur J Surg Oncol (2017) 43(9):1768–75. doi: 10.1016/j.ejso.2017.05.019 28602173

[12] Zhu Y Zhang SL Ye DW Yao XD Jiang ZX Zhou XY. Predicting Pelvic Lymph Node Metastases in Penile Cancer Patients: A Comparison of Computed Tomography, Cloquet’s Node, and Disease Burden of Inguinal Lymph Nodes. Onkologie (2008) 31(1-2):37–41. doi: 10.1159/0000112462 18268397

[13] Zhu Y Zhang SL Ye DW Yao XD Dai B Zhang HL. Prospectively Packaged Ilioinguinal Lymphadenectomy for Penile Cancer: The Disseminative Pattern of Lymph Node Metastasis. J Urol (2009) 181(5):2103–8. doi: 10.1016/j.juro.2009.01.041 19286211

[14] Lughezzani G Catanzaro M Torelli T Piva L Biasoni D Stagni S. The Relationship Between Characteristics of Inguinal Lymph Nodes and Pelvic Lymph Node Involvement in Penile Squamous Cell Carcinoma: A Single Institution Experience. J Urol (2014) 191(4):977–82. doi: 10.1016/j.juro.2013.10.140 24262497

[15] Li Z Guo S Wu Z Han H Li Z Wang Y. Subclassification of Pn3 Staging Systems for Penile Cancer: Proposal for Modification of the Current TNM Classification. Urol Oncol (2017) 35(9):543.e1–.e6. doi: 10.1016/j.urolonc.2017.04.009 28578871

[16] Yao K Tu H Li YH Qin ZK Liu ZW Zhou FJ. Modified Technique of Radical Inguinal Lymphadenectomy for Penile Carcinoma: Morbidity and Outcome. J Urol (2010) 184(2):546–52. doi: 10.1016/j.juro.2010.03.140 20620415

[17] Yao K Zou ZJ Li ZS Zhou FJ Qin ZK Liu ZW. Fascia Lata Preservation During Inguinal Lymphadenectomy for Penile Cancer: Rationale and Outcome. Urology (2013) 82(3):642–7. doi: 10.1016/j.urology.2013.05.021 23876593

[18] Liu JY Li YH Zhang ZL Yao K Ye YL Xie D. The Risk Factors for the Presence of Pelvic Lymph Node Metastasis in Penile Squamous Cell Carcinoma Patients With Inguinal Lymph Node Dissection. World J Urol (2013) 31(6):1519–24. doi: 10.1007/s00345-013-1024-4 23455885

[19] Lont AP Kroon BK Gallee MP van Tinteren H Moonen LM Horenblas S. Pelvic Lymph Node Dissection for Penile Carcinoma: Extent of Inguinal Lymph Node Involvement as an Indicator for Pelvic Lymph Node Involvement and Survival. J Urol (2007) 177(3):947–52. doi: 10.1016/j.juro.2006.10.060 17296384

[20] Yao K Chen Y Ye Y Wu Z Chen D Han H. Lymph Node Mapping in Penile Cancer Patients Undergoing Pelvic Lymph Node Dissection. J Urol (2020) 205(1):145–51. doi: 10.1097/JU.0000000000001322 32755338

[21] Carver BS Cronin AM Eggener S Savage CJ Motzer RJ Bajorin D. The Total Number of Retroperitoneal Lymph Nodes Resected Impacts Clinical Outcome After Chemotherapy for Metastatic Testicular Cancer. Urology (2010) 75(6):1431–5. doi: 10.1016/j.urology.2009.11.076 PMC3654386 20299079

[22] Graafland NM van Boven HH van Werkhoven E Moonen LM Horenblas S. Prognostic Significance of Extranodal Extension in Patients With Pathological Node Positive Penile Carcinoma. J Urol (2010) 184(4):1347–53. doi: 10.1016/j.juro.2010.06.016 20723934

[23] Zargar-Shoshtari K Djajadiningrat R Sharma P Catanzaro M Zhu Y Nicolai N. Establishing Criteria for Bilateral Pelvic Lymph Node Dissection in the Management of Penile Cancer: Lessons Learned From an International Multicenter Collaboration. J Urol (2015) 194(3):696–701. doi: 10.1016/j.juro.2015.03.090 25801766

[24] Zhang ZL Yu CP Liu ZW Velet L Li YH Jiang LJ. The Importance of Extranodal Extension in Penile Cancer: A Meta-Analysis. BMC Cancer (2015) 15:815. doi: 10.1186/s12885-015-1834-4 26510975 PMC4625878

[25] de Carvalho JP Patricio BF Medeiros J Sampaio FJ Favorito LA. Anatomic Aspects of Inguinal Lymph Nodes Applied to Lymphadenectomy in Penile Cancer. Adv Urol (2011) 2011:952532. doi: 10.1155/2011/952532 22110493 PMC3205725

[26] Zargar-Shoshtari K Sharma P Djajadiningrat R Catanzaro M Ye DW Zhu Y. Extent of Pelvic Lymph Node Dissection in Penile Cancer may Impact Survival. World J Urol (2016) 34(3):353–9. doi: 10.1007/s00345-015-1593-5 26026817

[27] Azizi M Aydin AM Hajiran A Lai A Kumar A Peyton CC. Systematic Review and Meta-Analysis-Is There a Benefit in Using Neoadjuvant Systemic Chemotherapy for Locally Advanced Penile Squamous Cell Carcinoma? J Urol (2020) 203(6):1147–55. doi: 10.1097/JU.0000000000000746 31928407

