# Supplementary material for: Deep Inguinal Lymph Node Metastases Can Predict Pelvic Lymph Node Metastases and Prognosis in Penile Squamous Cell Carcinoma
Source: Front Oncol. 2021 Sep 15;11:715799. doi: 10.3389/fonc.2021.715799 (PMC8479104; doi:10.3389/fonc.2021.715799)
Supplement: Supplementary Table 1 — Pelvic lymph node metastasis based on inguinal lymph node characteristics. ILN, inguinal lymph node; ENE, extranodal extension; PLNM, pelvic lymph node metastasis. [file Table_1.docx]

**Supplementary Table 1.** Pelvic lymph node metastasis based on inguinal lymph node characteristics

| No. of positive ILN | | ILN pathological characteristics | | | | | | | | |
| --- | --- | --- | --- | --- | --- | --- | --- | --- | --- | --- |
|  |  | Deep ILN | |  | ENE | |  | Side involvement | | Total |
|  |  | Negative | Positive |  | Absent | Present |  | Unilateral | Bilateral |  |
| 1 | All | 55 | - |  | 38 | 17 |  | 77 | - | 55 |
|  | PLNM | 7 | - |  | 3 | 4 |  | 7 | - | 7 |
|  | PLNM rate | 13% | - |  | 8% | 24% |  | 13% | - | 13% |
|  |  |  |  |  |  |  |  |  |  |  |
| 2 | All | 47 | 4 |  | 40 | 11 |  | 33 | 18 | 51 |
|  | PLNM | 6 | 3 |  | 5 | 4 |  | 7 | 2 | 9 |
|  | PLNM rate | 13% | 75% |  | 13% | 36% |  | 21% | 11% | 18% |
|  |  |  |  |  |  |  |  |  |  |  |
| 3 | All | 23 | 8 |  | 13 | 18 |  | 9 | 22 | 31 |
|  | PLNM | 5 | 4 |  | 3 | 6 |  | 2 | 7 | 9 |
|  | PLNM rate | 22% | 50% |  | 23% | 33% |  | 22% | 32% | 29% |
|  |  |  |  |  |  |  |  |  |  |  |
| 4 | All | 16 | 7 |  | 7 | 16 |  | 8 | 16 | 23 |
|  | PLNM | 4 | 5 |  | 2 | 7 |  | 3 | 7 | 9 |
|  | PLNM rate | 25% | 71% |  | 29% | 44% |  | 38% | 44% | 39% |
|  |  |  |  |  |  |  |  |  |  |  |
| 5 | All | 8 | 3 |  | 6 | 5 |  | 1 | 10 | 11 |
|  | PLNM | 2 | 3 |  | 2 | 3 |  | - | 5 | 5 |
|  | PLNM rate | 25% | 100% |  | 33% | 60% |  | - | 50% | 45% |
|  |  |  |  |  |  |  |  |  |  |  |
| ≥6 | All | 7 | 11 |  | 11 | 7 |  | 2 | 15 | 18 |
|  | PLNM | 4 | 10 |  | 7 | 7 |  | 2 | 12 | 14 |
|  | PLNM rate | 57% | 91% |  | 64% | 100% |  | 100% | 80% | 78% |
|  |  |  |  |  |  |  |  |  |  |  |
| Total | All | 156 | 33 |  | 115 | 74 |  | 109 | 80 | 189 |
|  | PLNM | 28 | 25 |  | 22 | 31 |  | 21 | 32 | 53 |
|  | PLNM rate | 18% | 76% |  | 19% | 42% |  | 19% | 40% | 28% |

ILN: inguinal lymph node; ENE: extranodal extension; PLNM: pelvic lymph node metastasis
