# Supplementary figures and images for: Deep Inguinal Lymph Node Metastases Can Predict Pelvic Lymph Node Metastases and Prognosis in Penile Squamous Cell Carcinoma
Source: Front Oncol. 2021 Sep 15;11:715799. doi: 10.3389/fonc.2021.715799 (PMC8479104; doi:10.3389/fonc.2021.715799)

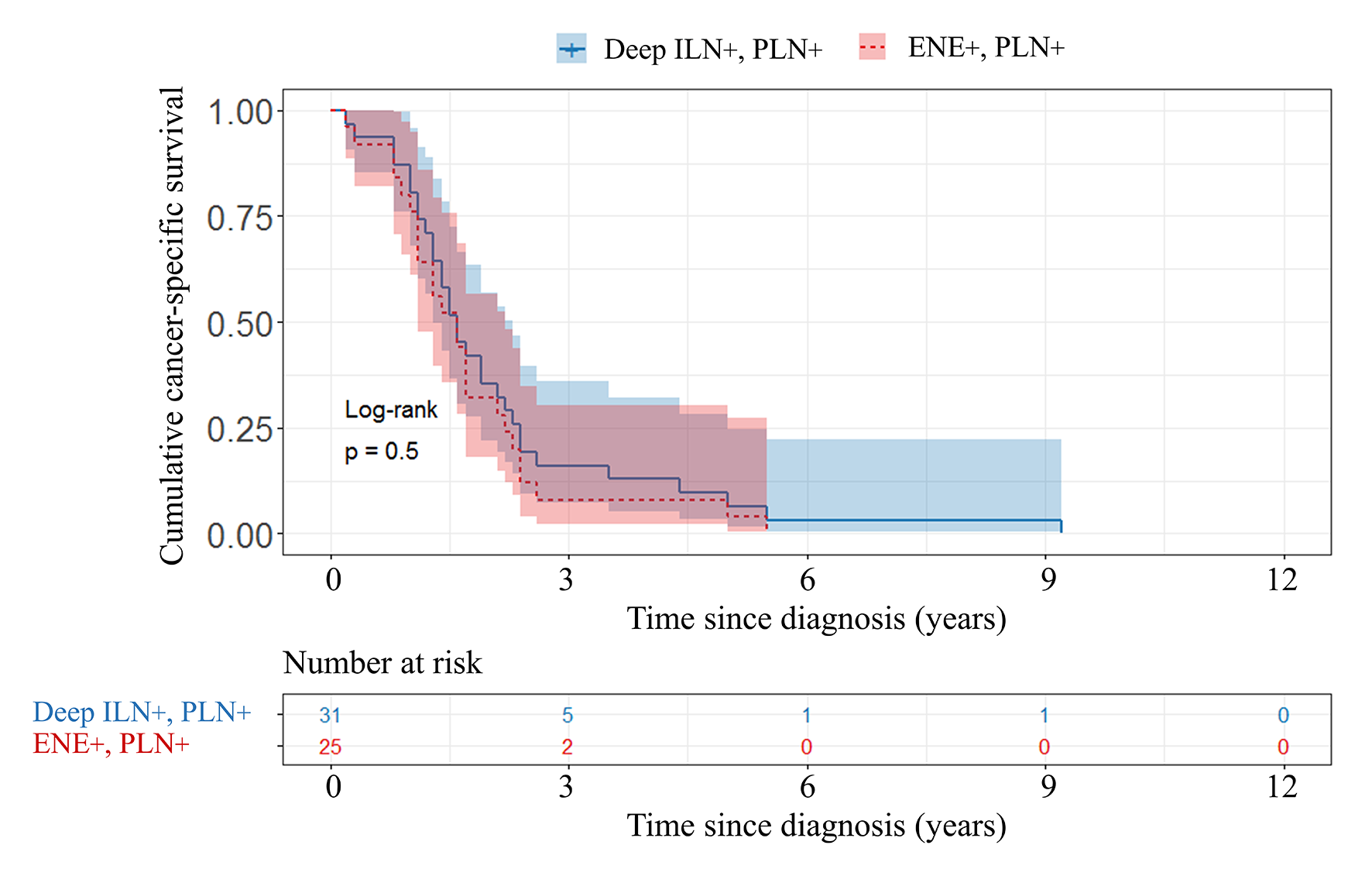

Supplement: Supplementary Figure 1 — Kaplan-Meier CSS curve of patients with deep ILNM, ENE and PLNM. CSS, cancer-specific survival; PLN, pelvic lymph node; ILN, inguinal lymph node; ENE, extranodal extension. [file Image_1.tif]
